# Whole breast radiotherapy in cN0 early breast cancer patients with pathological sentinel lymph nodes (pN1mic, pN1a) without axillary dissection: preliminary results of the observational LISEN trial

**DOI:** 10.1007/s00066-022-01944-z

**Published:** 2022-05-02

**Authors:** Lucia Anna Ursini, Marianna Nuzzo, Consuelo Rosa, Marzia Borgia, Luciana Caravatta, Monica Di Tommaso, Marianna Trignani, Fiorella Cristina Di Guglielmo, Giampiero Ausili Cefaro, Domenico Angelucci, Marzia Muzi, Gianluigi Martino, Ettore Cianchetti, Simona Grossi, Saveria Tavoletta, Davide Brocco, Antonino Grassadonia, Nicola Tinari, Simona Gildetti, Nicola D’Ostilio, Liborio Stuppia, Annamaria Porreca, Marta Di Nicola, Domenico Genovesi

**Affiliations:** 1 Department of Radiation Oncology, SS. Annunziata Hospital, “G. D’Annunzio” University of Chieti, Via dei Vestini, 66100 Chieti, Italy; 2 Department of Neuroscience, Imaging and Clinical Sciences, “G. D’Annunzio” University of Chieti, Via dei Vestini, 66100 Chieti, Italy; 3 Department of Pathological Anatomy, ASL 02 Lanciano-Vasto-Chieti, Via dei Vestini, 66100 Chieti, Italy; 4 Division of Breast Radiology, Department of Radiology, G. Bernabeo Hospital, Contrada Santa Liberata, 66026 Ortona, Chieti, Italy; 5 Department of Radiological Sciences, Institute of Nuclear Medicine, SS. Annunziata Hospital, Via dei Vestini, 66100 Chieti, Italy; 6 Division of Surgical Senology, G. Bernabeo Hospital, Contrada Santa Liberata, 66026 Ortona, Chieti, Italy; 7 Clinical Oncology Unit, SS. Annunziata Hospital, Via dei Vestini, 66100 Chieti, Italy; 8 Department of Medical, Oral and Biotechnological Sciences and CeSI-MeT, “G. D’Annunzio” University of Chieti, Via dei Vestini, 66100 Chieti, Italy; 9 Department of Oncology, Floraspe Renzetti Hospital, Via per Fossacesia 1, Lanciano, 66034 Chieti, Italy; 10 Center for Advanced Studies and Technology (CAST), “G. D’Annunzio” University of Chieti, Via dei Vestini, 66100 Chieti, Italy; 11 Department of Economics, “G. D’Annunzio” University of Chieti-Pescara, Pescara, Italy; 12 Laboratory of Biostatistics, Department of Medical, Oral and Biotechnological Sciences, “G. D’Annunzio” University of Chieti, Via dei Vestini, 66100 Chieti, Italy

**Keywords:** Axillary surgery omission, Breast cancer, Conservative surgery, Radiotherapy, Sentinel lymph node

## Abstract

**Purpose:**

Axillary management remains unclear when sentinel lymph node (SLN) results are positive in cN0 patients with breast cancer (BC). The trial ACOSOG Z0011 represented a revolution with axillary lymph node dissection (ALND) omission in SLN+ patients, despite critiques regarding non-uniformity of radiation fields. We conducted an observational study (LISEN) where whole breast radiotherapy (WBRT) was planned with tangential fields without nodal irradiation in patients eligible for the Z0011 trial.

**Methods:**

Inclusion criteria were female patients with histologically proven BC, cT1-2cN0, planned conservative surgery, no neoadjuvant therapy. Patients were stratified into two groups: micrometastatic (pN1mic, group 1) and macrometastatic (pN1a, group 2) lymph nodes. Tangential field WBRT was mandatory. Clinical outcomes were analysed, measured from surgery until the first event.

**Results:**

In all, 199 patients underwent conservative surgery and SLN biopsy; 133 patients meeting criteria were analysed: 41 patients (30.8%) pN1mic and 92 (69.2%) pN1a. The 5‑year disease-free survival (DFS) was 95.0% (85.9–100%) in group 1 and 93.0% (86.3–100.0%) in group 2 (*p* = 0.78). Overall survival (OS) was 100% (100–100%) in group 1 and 97.4% (92.4–100%) in group 2 (*p* = 0.74). For the whole cohort DFS and OS were 93.6% (88.2–99.4%) and 96.9% (91.5–100.0%), respectively. For groups 1 and 2, the 5‑year outcomes were 5.0% (0.0–14.4%) and 2.3% (0.0–6.1%) for local recurrence (*p* = 0.51), and 6.2% (0.0–17.4%) and 7.0% (0.0–13.7%) for distant metastasis (*p* = 0.61), respectively. In group 1, regional recurrence (RR) and local regional recurrence (LRR) were 5.0% (0.0–14.1%; *p* = 0.13). In group 2, RR and LRR were 0.0% (0.0–0.0%).

**Conclusion:**

Our results showed good regional control in patients who met the Z0011 trial criteria. WBRT, without nodal surgery or RT, avoiding axillary morbidity, seems to be a good choice.

## Introduction

In the context of more conservative surgical techniques, axillary management is constantly evolving in breast cancer (BC) treatment. In the past, axillary lymph node dissection (ALND) was always performed as a tumour staging procedure and to improve regional control (RC) [1]. However, ALND may cause significant complication, such as lymphoedema, seroma and infection, with potential impairment of upper limb motion [2, 3].

Sentinel lymph node (SLN) biopsy was introduced in the early 1990s to avoid ALND for negative lymph nodes [4]. In comparison to ALND, SLN technique may yield fewer side effects, allowing an approximately three-fold reduction in the incidence of lymphoedema [5]. Hence, SLN biopsy became an alternative to ALND in patients with clinically (cN0) and pathologically (pN0) node-negative stage. Otherwise, the optimal management of the SLN has long been debated when a clinical negative node (cN0) result is pathologically positive (pN1) for the likelihood of additional axillary node metastases [6].

In this scenario, the randomized clinical trial American College of Surgeons Oncology Group Z0011 (ACOSOG Z0011) was conducted to determine whether overall survival (OS) of pathologically positive node patients treated with breast conserving surgery (BCS) and SLN biopsy alone was noninferior to OS in women treated with ALND. All patients had a planned lumpectomy, tangential whole breast radiotherapy (WBRT) and adjuvant systemic therapy, without nodal irradiation. The authors concluded that ALND could be safely omitted with good results in OS [3]. Similar promising results were confirmed in more recent prospective studies, with good rates of OS or disease-free survival (DFS) [7, 8]. The ACOSOG trial received several critiques, especially regarding the nonuniformity of RT treatment fields and the lack of RT records, which were available for approximately one-third of treated patients [9]. However, since 2011, this study represented a revolution in daily surgical practice and in currently guidelines, although the optimal radiation approach had still not been defined and there were several uncertainties [10–13].

The objective of this study is to report the preliminary results of the “LInfonodo SENtinella” (LISEN) trial, an observational study aimed to confirm the long-term efficacy of ALND omission, in terms of local recurrence (LR), regional recurrence (RR), local and regional recurrence (LRR), distant metastasis (DM), DFS and OS, in cT1‑2 cN0 M0 patients undergoing conservative surgery with 1 or 2 positive SLN. In this study, the WBRT was always planned with tangential fields without nodal irradiation.

## Patients and methods

The study was designed as a prospective observational study by the Interdisciplinary Group for Oncological Care of the EUSOMA Breast Center of Lanciano–Vasto–Chieti ASL and was definitively approved on the 24 January 2013 by the Ethics Committee of the “SS. Annunziata” Hospital, “G. d’Annunzio” University, Chieti, Italy.

### Patient characteristics

LISEN trial is a cohort, prospective, observational study. From January 2013 to June 2019 all consecutive eligible patients with BC were enrolled for the LISEN trial in the Division of Breast Surgery in Ortona (Chieti, Italy). All patients provided written informed consent.

Inclusion criteria were histologically proven female BC patients, staged as cT1‑2 cN0 M0 with eligible criteria for BCS. Exclusion criteria were cT3–4, cN+ in clinical and/or radiological assessment, positive lymph nodes biopsy before surgery, neoadjuvant chemotherapy or endocrine therapy, previous BC, active collagenopathies that contraindicate RT, intraoperative or postoperative evidence of extracapsular extension on SLN.

Patients were divided into two groups according to micrometastatic (group 1) and macrometastatic (group 2) lymph nodes status. Isolated tumour cells (pN0i+) were not considered in our analysis.

### Surgery

All patients underwent BCS. SLN biopsy was performed after preoperative lymphoscintigraphy by injection of technetium (99mTc)–sulphur colloid into the skin, subdermally or in the peritumoral area of the breast before surgery. Positive lymph nodes were classified according to their dimensions: micrometastasis (pN1mic: size > 0.2 mm and not more than 2.0 mm) and macrometastasis (pN1 mac: size > 2 mm) detected with haematoxylin–eosin staining or immunohistochemical analysis with antibodies to cytokeratin.

Patients with no more than two micrometastatic or metastatic SLN in the absence of other clinical and radiological suspected axillary lymph nodes and no evidence of extracapsular extension in the removed lymph nodes did not undergo ALND.

### Systemic therapy

Adjuvant systemic therapy was planned after surgery according to the characteristics of the patient (age, menopausal status, possible comorbidities) and the tumour (tumour size, lymph node status, biological characteristics of the neoplasm, such as, receptor structure, HER2 expression, proliferative activity, gene profiles) [11, 12]. The biological characteristics of the tumour evaluated in immunohistochemistry allow to distinguish 5 different types of neoplasms: luminal A (hormone receptor positive, Ki67 < 14%, HER2 negative), luminal B (hormone receptor positive, Ki67 ≥ 14%, HER2 negative), luminal B HER2 positive (hormone receptor positive, any Ki67, HER2 positive), HER2 over-expressing (hormone receptor negative and HER2 positive) and triple negative (hormone receptors and HER2 negative), with different biological behaviour and sensitivity to endocrine therapy and chemotherapy [11, 12].

Chemotherapy treatment, represented mostly by anthracyclines followed by taxanes, started possibly within 30 days of surgery. The addition of trastuzumab in patients with HER2-positive cancer was administered concomitantly with chemotherapy containing taxanes and then extended to complete 1 year. Adjuvant endocrine therapy was indicated in all patients with positive hormone receptors for 5 years and, if combined with chemotherapy, started at the end of the latter [11, 12].

### Radiotherapy

A computed tomography (CT) simulation was performed in supine position (slices thickness = 0.5 cm), with a breast board immobilization to ensure treatment reproducibility. External markers were placed at the time of CT simulation: wire markers were used to identify breast and tumour bed.

The clinical target volume (CTV) included the apparent/total CT glandular breast tissue, and in its dorsal border excluded the pectoralis major muscles, the external aspect of the ribs and chest wall/intercostal muscles. The superior border usually extended up to the level of inferior margin of the sternoclavicular joint. The lowest CT slice where the breast was still visible was considered the inferior border. The medial border was marked at the ipsilateral edge of the sternum. Surgical clips placed at the lumpectomy site allowed tumour bed identification [14].

The planning target volume (PTV) included the whole breast soft tissues from 5 mm below the skin surface to the deep fascia, including the pectoralis major muscle.

Doses ranged from 40 Gy to 50 Gy (2.0–2.5 Gy/fraction) to the PTV; 4 Gy to 10 Gy (0.25–2 Gy/fraction) were used for the concomitant/sequential boost to the tumour bed, as internal protocol, corresponding to the surgical clips if present.

Organs at risk (OARs), identified according to the tumour side, were the ipsilateral lung and humeral head; the whole heart or the liver in case of left or right breast irradiation, respectively. According with Quantitative Analyses of Normal Tissue Effects in the Clinic (QUANTEC) criteria, dose constraints respected as following: for left-side irradiation, whole heart V_5_ ≤ 40–50%, V_20_ < 12.5%, V_25_ < 10% and median heart dose ≤ 5 Gy; ipsilateral lung V_5_ < 40%, V_20_ ≤ 15%, V_30_ ≤ 10% and median lung dose ≤ 8–9 Gy; ipsilateral humeral head V_50_ < 5%, Liver V_40_ < 30% and D_mean_ < 26 Gy.

All patients received 3D-CRT with standard or field in field WBRT to treat the whole breast without nodal irradiation. Electrons or photons were used for boost. Portal images were routinely scheduled for the first 3 days of treatment and then weekly to verify the correct patient positioning before treatment delivery.

In case of adjuvant chemotherapy, radiotherapy was started a month after the end of the systemic therapy.

### Follow-up

All patients underwent oncological follow-up according to international guidelines: mammography and breast ultrasound after 6 months after the end of RT and then annually, clinical breast examination and axillary ultrasound every 6 months from the date of surgery for the first 36 months and thereafter annually. Medical oncologists managed adjuvant systemic therapy (hormone therapy and/or trastuzumab) and prescribed blood exams, tumoral markers, chest x‑ray and abdominal ultrasound for the first 36 months and thereafter annually. Radiation oncologists recorded all follow-up exams and any locoregional toxicity. All recurrences and/or distant metastases were recorded.

Local and regional recurrence were divided into breast relapse defined as LR, lymph-node relapse as RR and both breast and nodal relapse as LRR. Distant metastasis (DM) was defined as clinical evidence of distant disease based on clinical and/or radiographic findings. DFS was defined as the time between surgery and the first evidence of clinical and/or radiographic recurrence (local and regional recurrence or distant metastasis) or death from any cause. OS was defined as the time interval between surgery and death.

### Statistical analysis

Descriptive statistics included frequencies and proportions for categorical variables, median (range) for continuous variables. The Mann–Whitney *U*-test was used to assess statistically significant age differences between the two groups (pN1mic = micrometastasis and pN1a = macrometastasis). The χ^2^ test was used to detect associations between the groups and the categorical variables. We analysed all time-to-event distributions using the Kaplan–Meier method to calculate the probability of LR, RR, LRR, DM, DFS and OS rates at 5 years. The follow-up time was defined as the time interval between the date of surgery and the date of the first verified event according to the definition for each clinical endpoint. For patients in which no event occurred, we defined the follow-up time interval as the time elapsed until the last scheduled follow-up visit.

Lastly, the log rank test was used to compare the survival distribution between groups. All statistical tests were 2‑sided, with the significance level set at *p* < 0.05. Analyses were performed using the R software environment for statistical computing and graphics (version 3.4.1; http://www.r-project.org).

## Results

**Fig. 1:**
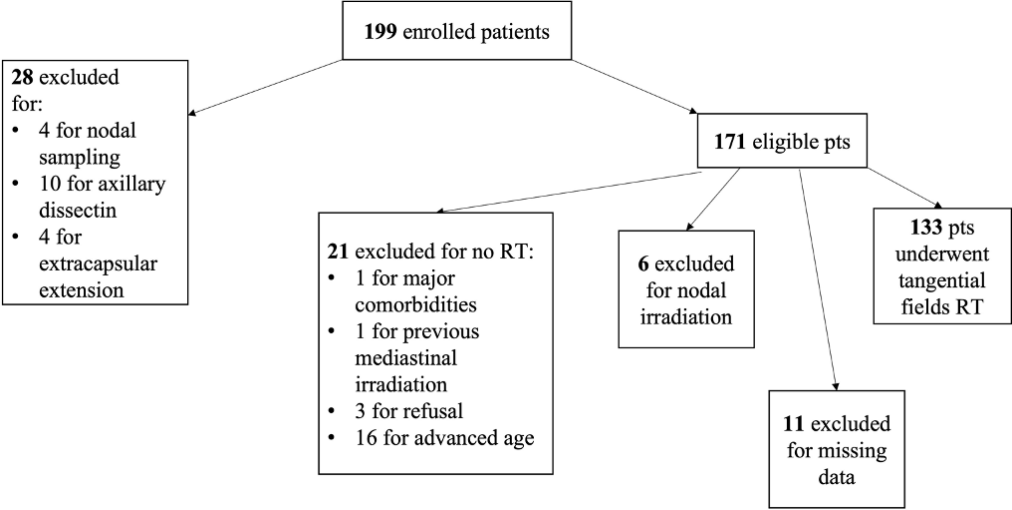
Flowchart of patients of the LISEN trial. *pts* patients, *RT* radiotherapy

In total, as depicted in Fig. 1, 133 patients met our inclusion criteria in the median age of 56 years (range 35–83 years) and were divided into two groups, according to their lymph node status: 41 patients (30.8%) in the micrometastatic (group 1) and 92 (69.2%) in the macrometastatic (group 2). Patients, tumour and treatment characteristics were summarized in Table 1.

**Table 1:** Patients and tumour characteristics in 133 breast cancer subjects with micrometastatic and macrometastatic sentinel lymph node. Data are expressed as frequencies and proportions for categorical variables, median (range) for continuous variables

	All patients (groups 1 and 2) *n* (%)	Micrometastases (group 1) *n* (%)	Macrometastases (group 2) *n* (%)	χ^2^ test *p*-value for groups 1 and 2
	133 (100%)	41 (30.8%)	92 (69.2%)	
**Age (years), ***median (range)*	56 (35–83)	51 (35.0–75.0)	58 (38.0–83.0)	0.019^a^
**Surgery**				
Lumpectomy	109 (81.9%)	33 (80.5)	76 (82.6)	–
Quadrantectomy	24 (18.1%)	8 (19.5)	16 (17.4)	–
**Pathological stage**				
Tis	1 (0.8)	0 (0.0)	1 (1.1)	0.286
T1a	1 (0.8)	0 (0.0)	1 (1.1)	
T1b	28 (21.1)	10 (24.4)	18 (19.6)	
T1c	80 (60.1)	23 (56.1)	57 (61.9)	
T2	23 (17.2)	8 (19.5)	15 (16.3)	
**Histological type**				
Ductal	109 (82.0)	36 (87.8)	73 (79.3)	0.823
Lobular	10 (7.5)	1 (2.4)	9 (9.8)	
Other	14 (10.5)	4 (9.8)	10 (10.9)	
**Grade**				
1	71 (53.4)	22 (53.7)	49 (53.3)	0.818
2	54 (40.6)	15 (36.6)	39 (42.4)	
3	8 (6.0)	4 (9.8)	4 (4.3)	
**Molecular subtypes**				
Luminal A	88 (66.2)	28 (68.3)	60 (65.2)	0.113
Luminal B (HER2 negative)	30 (22.5)	8 (19.5)	22 (23.9)	
Luminal B (HER2 positive)	10 (7.5)	3 (7.3)	7 (7.6)	
HER2+	1 (0.8)	0 (0.0)	1 (1.1)	
Basal-like	4 (3.0)	2 (4.9)	2 (2.2)	
**Nodal ratio**				
1/1	26 (19.5)	12 (29.3)	14 (15.2)	0.627
1/2	52 (39.1)	14 (34.1)	38 (41.3)	
1/3	24 (18.0)	7 (17.1)	17 (18.5)	
1/4	11 (8.3)	5 (12.2)	6 (6.5)	
1/5	1 (0.8)	0 (0.0)	1 (1.1)	
2/2	8 (6.0)	2 (4.9)	6 (6.5)	
2/3	8 (6.0)	1 (2.4)	7 (7.6)	
2/4	2 (1.5)	0 (0.0)	2 (2.2)	
2/5	1 (0.8)	0 (0.0)	1 (1.1)	
**Adjuvant systemic therapy**				
Chemotherapy	55	10	45	–
Trastuzumab	11	2	9	–
Endocrine therapy	126	38	88	–

^a^*p*-value derived from Whitney–Mann *U*-test

### Surgery and pathological characteristics

All 133 patients underwent BCS: 109 patients (81.9%) underwent lumpectomy, 24 (18.1%) quadrantectomy. Negative surgical margins were obtained in all patients.

SLN biopsy was performed with a median total number of removed lymph nodes of 2 (range 1.0–4.0). The median number of histologically positive nodes was 1 (range 1.0–2.0).

As reported in Table 1, patients were divided in two groups, according their lymph nodes status: 41 patients (30.8%) in the micrometastatic, group 1, and 92 (69.2%) in the macrometastatic, group 2.

### Adjuvant treatment

Adjuvant systemic therapy was administered according to staging and tumour biology. Fifty-seven patients underwent adjuvant chemotherapy, 11 biological therapy (trastuzumab) and endocrine therapy was administered to 126 patients.

Of the 133 patients undergoing tangential fields WBRT, 126 (94.7%) received conventional fractionation (50 Gy in 25 fraction) and 7 (5.3%) hypofractionation (from 40 to 40.05 Gy in 16–15 fraction); 128 (96.2%) received a boost. A sequential boost on the tumour bed was prescribed for 126 patients: 9 Gy in 2 patients (1.5%) and 10 Gy in 124 (93.2%). Two patients (1.5%) had a concomitant boost: 0.25 Gy/day in 16 fractions, for a total dose of 4 Gy.

### Outcomes

The median follow-up was 50 months (range 5.0–91.0 months). The 5‑year outcomes of the whole cohort were 3.2% (0.0–7.4%) for LR, for RR and LRR the rate was 1.5% (0.0–4.4%), DM was 6.7% (0.6–12.3%), DFS was 93.6% (88.2–99.4%) and OS was 96.9% (91.5–100.0%).

Table 2 reports the characteristics of patients developing locoregional and distant metastases.

**Table 2:** Characteristics of LISEN patients with local recurrence (LR), regional recurrence (RR), distant metastasis (DM) and death

Pts	Age	Pathological Stage		G	Molecular Subtype	HER‑2	Ki67 (in %)	LR	RR	DM Site and timing	Death
1	41	T1c	N1a	G1	Luminal B	Positive	30	Yes	No	No	No
2	42	T1c	N1mic	G1	Luminal A	Negative	5	Yes	No	No	No
3	45	T2	N1mic	G2	Luminal B	Negative	70	Yes	Yes (S)	Bone (M)	No
4	47	T1c	N1a	G1	Luminal A	Negative	20	No	No	Bone, liver, lung	No
5	48	T1b	N1a	G2	Luminal B	Negative	40	Yes	No	Bone (M)	No
6	49	T1c	N1a	G2	Luminal B	Negative	60	No	No	Bone	Yes
7	56	T2	N1a	G1	Luminal B	Positive	12	No	No	Bone	No

*pts* patients, *G* grading, *DM* distant metastases, *LR* local recurrence, *RR* regional recurrence, *S* synchronous, *M* metachronous

**Fig. 2:**
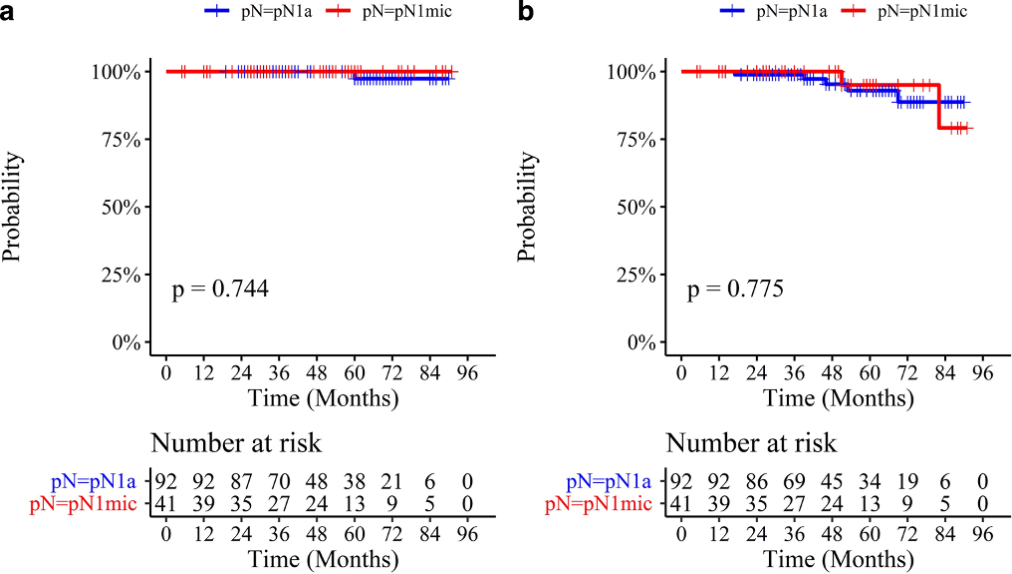
Kaplan–Meier curves adjusted for age for pN groups evaluated for overall survival (OS, **a**) and disease-free survival (DFS, **b**). *P*-values for difference between two groups of curves were calculated by the log rank test

Fig. 2 reports Kaplan–Meier curves adjusted for age for DFS and OS in both groups. The log-rank test confirms not significant differences between groups. In particular, the 5‑year outcomes of BC patients stratified for groups were DFS 95.0% (85.9–100%) in group 1 and 93.0% (86.3–100.0%) in group 2 (*p* = 0.78). OS was 100% (100–100%) in group 1 and 97.4% (92.4–100%) in group 2 (*p* = 0.74).

For LR the 5‑year outcomes of BC patients stratified for groups were 5.0% (0.0–14.4%) in group 1 and 2.3% (0.0%–6.1%) in group 2 (*p* = 0.51). In group 1, RR and LRR were 5.0% (0.0–14.1%; *p* = 0.13); in group 2, RR and LRR were 0.0% (0.0–0.0%). Indeed, for DM we have 6.2% (0.0–17.4%) in group 1 and 7.0% (0.0–13.7%) in group 2 (*p* = 0.61).

## Discussion

We conducted the LISEN trial with the aim to evaluate LR, RR, LRR, DM, DFS and OS in BC patients with one or two metastatic SLN, without nodal surgery neither nodal RT, in de-escalation strategy, in patients with ACOSOG Z0011 criteria [2, 3], achieving excellent local, regional and distant disease control. The risk of disease recurrence seems not to increase with ALND and nodal irradiation omissions.

Over the last few years, breast surgery and management of the axilla has become more conservative [4]. The increased use of screening mammography in the 1990s contributed to decrease axillary involvement by up to 22% [15]. Furthermore, in clinical practice, SLN biopsy represents a routine approach, allowing ALND omission in patients with clinical negative SLN. In case of pathological negative SLN, axillary recurrence occurs in about 1% of patients [16].

The NSABP B‑32 enrolled 5611 BC patients with clinically negative nodes. Their analysis was conducted only on node negative patients: 1978 in SLN resection plus ALND group (group 1) and 2011 in SLN resection alone group (group 2). The authors reported no statistically significant difference in OS (*p* = 0.12), DFS (*p* = 0.54) and regional control (*p* = 0.55), respectively, with fewer side effects in the second group. The NSABP B‑32 trial demonstrated no statistically significant difference in survival between ALND and SLN surgery alone in patients with negative SLN [17].

From 1999–2001, several patients were enrolled in different studies evaluating ALND omission in positive SLN. The phase III IBCSG, the AMAROS and the ACOSOG Z0011 trials reported first results comparing clinical outcomes of patients who underwent SLN biopsy respect to ALND.

The multicenter IBCSG 23-01 trial evaluated the impact of micrometastatic SLN, comparing the non-ALND group with the ALND group. Patients receiving breast-conserving surgery received WBRT or intraoperatively partial breast irradiation. Only 5% of these patients was treated with axillary lymph nodes RT. The 5‑year DFS was 87.8% in the non-ALND group and 84.4% in the ALND group (*p* = 0.16): the DFS in the non-ALND group was non-inferior to the ALND group (*p* = 0.0042). Therefore, ALND should be avoided when the sentinel node is minimally involved, reducing axillary surgery morbidity with no impact on survival [18]. These results were confirmed at 10 years with a DFS of 76.8% in the non-ALND group and 74.9% in the ALND group (*p* = 0.24; *p* = 0.0024 for non-inferiority) [19].

The non-inferiority of axillary radiation treatment respect to ALND in regional control, with fewer side-effects, was confirmed in the multicenter AMAROS trial.

Positive sentinel node occurred in 1425 patients; 744 of them had been assigned to ALND and 681 to axillary radiotherapy. All three levels of the axilla and the medial part of the supraclavicular fossa were included in radiation treatment. Axillary recurrence occurred in 4 patients in the ALND group and 7 in the axillary radiotherapy group. The 5‑year axillary recurrence was 0.43% for the ALND group versus 1.19% for the axillary radiotherapy group. The 5‑year DFS and OS were 86.9 and 82.7% (*p* = 0.18) and 93.3 and 92.5% (*p* = 0.34), respectively, with no statistically significant differences between the two groups. The authors concluded that both ALND and axillary radiotherapy could provide excellent and comparable axillary control in positive SLN, with significantly less morbidity in case of RT [20].

The ACOSOG Z0011 trial, where cT1‑2 N0 M0 BC patients were treated with BCS and SLN, aimed to demonstrate the impact of ALND omission on OS in patients with SLN metastases treated with lumpectomy, adjuvant systemic therapy and tangential field RT without a third field nodal irradiation. Of the 891 patients enrolled, 445 were randomized in the ALND group and 446 in the SLN-alone group [6]. Preliminary results, with a median follow-up of 6.3 years, demonstrated that the use of SLN alone did not negatively impact both 5‑year OS and DFS compared with ALND group (92.5 vs 91.8%, *p* = 0.008; and 83.9 vs 82.2%, *p* = 0.14, respectively) [2]. Ten-year results confirmed the non-inferiority of SLN compared to ALND, both in term of DFS (86.3 vs 83.6%, *p* = 0.02) and OS rate (80.2 vs 78.2%, *p* = 0.32), respectively [3].

However, the ACOSOG Z0011 trial received several critiques especially regarding the favourable selection bias (median age was 55 years, 70% had T1 tumours, 82% had oestrogen receptor-positive disease, 71% had only one positive SLN, and 44% had micrometastasis) and the variability in the extent and the administration of radiation treatments [9, 13]. Jagsi et al. tried to demonstrate that, although the Z0011 design did not contemplate nodal treatment, there were differences in radiation fields based on the extent of axillary surgery. Detailed radiation treatment records were obtained only for 228 patients in the ACOSOG trial, 124 in the SLN arm and 104 in the ALND arm. Most patients received tangential field RT alone: 103 patients (83.1%) and 82 (78.8%) in the SLN and ALND, respectively. Furthermore, 43 patients (18.9%) received directed regional nodal RT using ≥ three fields: 21 in the SLN arm and 22 in the ALND arm. There was no statistically significant difference between the two arms in the use of protocol-prohibited nodal RT fields, even if the treatment of a posterior axillary boost field was more common in patients who received SLN biopsy alone (*p* = 0.067). The authors concluded that, in the 228 patients analysed from the ACOSOG trial, most of them received tangential field RT alone, without significant differences in tangential field height between the two arms. Furthermore, it is worth considering additional nodal treatment in selected high-risk patients [9].

From these critiques, we conducted our observational study in a homogeneous population of cT1‑2 cN0 M0 patients treated with a radiotherapy fields uniformity, with only tangential RT without nodal irradiation, after SLN alone. Our preliminary results for DFS and OS rates in the SLN group are comparable to the ACOSOG Z0011 trial results (93.6 vs 83.9% and 96.9 vs 92.5%, respectively), in patients with similar favourable characteristics (pT1, positive hormonal receptors).

Concurrently, different studies have been conducted to verify the results of the ACOSOG study (Table 3). The prospective study of Kittaka et al. was conducted to confirm that the ACOSOG Z0011 criteria apply to Japanese early BC patients. The authors reported that the low RR rate could have been achieved by high tangent irradiation to all eligible patients and by adjuvant systemic therapy administration to most patients [7].

**Table 3:** Outcomes of studies evaluating sentinel lymph node (SLN) dissection and radiotherapy

Author (years)	Study type	SLN	Median follow-up (months)	Type of RT	5‑year LRR (in %)	5‑year DFS (in %)	5‑year OS (in %)
		SLN+ RT					
ACOSOG Z0011 (2011) [2]^a^	*P*	446	75.6 (62.4–84.0)	WBRT, High tangent, nodal RT	1.6	83.9	92.5
ACOSOG Z0011 (2017) [3]^a^	*P*	446	111.6 (82.8–124.0)	WBRT, High tangent, nodal RT	NA	80.2^b^	86.3^b^
Setton (2011) [21]	R	326	55.0 (1.0–158.0)	–	98.0^c,d^	95.0^c^	91.0^c^
		302		Supine or prone WBRT, High tangents, nodal RT	99.0^c,d^	96.0^c^	92.0^c^
Yi (2013) [22]	R	188	66.0 (14.4–134.4)	WBRT	NA	95.7	94.3
		121	64.8 (14.4–134.4)		NA	99.0	95.9
Wang (2014) [23]	R	1269^e^	73.0 (24.0–143.0)	Not specified	NA	NA	NA
		393			0.0	95.6	89.4
Morrow (2017) [8]	*P*	663	29.0 (2.0–76.0)	Supine or prone WBRT, nodal RT	NA	93.0	95.0
		484	37.0 (12.0–75.0)		1.0	NA	NA
Kittaka (2018) [7]	*P*	189	36.0 (10.0–64.0)	WBRT ± High tangent	1.1^f^	96.8^f^	NA
		183			NA	NA	NA
Jung (2019) [24]	R	–	–	Not specified	NA	NA	NA
		707			1.1	97.7	NA
LISEN trial^g^	*P*	131	44.0 (6.0–85.0)	WBRT	1.5	93.6	96.9

*SLN* sentinel lymph node dissection, *P* prospective, *R* retrospective, *RT* radiotherapy, *LRR* locoregional recurrence, *DFS* disease-free survival, *OS* overall survival, *WBRT* whole breast radiotherapy, *NA* not available

^a^SLN group

^b^10 years

^c^4 years

^d^Regional control

^e^SLND + ALND

^f^3 years

^g^SLN + RT group

The findings of ACOSOG Z0011 results were applied in a prospective study of 793 BC patients, cT1-2N0 with 1–2 positive SLN [8]. The 5‑year rate of breast-only recurrences, breast and nodal, and nodal and distant recurrence were 1.6, 0.7 and 0.7%, respectively. In particular, the authors examined a subset of 484 patients treated with SLN biopsy alone with known RT fields (21.0% prone breast RT, 58.0% supine tangent breast RT and 21% breast and nodal RT). At a median follow-up of 37 months, there were 5 nodal recurrences among this subgroup: 4 nodal and distant and 1 breast and axillary, with a 5-year cumulative rate of nodal recurrence of 1% [8].

Moreover, some retrospective studies reported clinical outcomes in patients with SLN biopsy alone or SLN biopsy followed by ALND, with or without specific information regarding types of RT (supine or prone WBRT, high tangents RT, nodal irradiation) [21–24]. Despite the heterogeneity of the analysed patients, clinical outcomes are favourable in patients treated with SLN biopsy alone followed by radiotherapy (Table 3; [21–24]).

It is well known as tumour size, grade, receptor status and different molecular profiles determined systemic therapy decisions, rather than the number of positive nodes. The ALND has a twofold role, i.e. for staging and cure. The ALND findings had always helped radiation oncologists in the decision of radiation treatment fields. The Z0011 trial publication made the optimal radiation fields for patients with positive SLNs who did not undergo ALND uncertain [13]. The lack of prospective data regarding field design led to consider some issues such as the probability of residual disease burden with the help of validated nomograms, in combination with clinical judgment. Tumour size, grade, histology, receptor status, lymphovascular invasion and number of positive SLN can be helpful in estimating the risk of additional positive nodes and guiding radiation field design.

In our study, most patients had positive oestrogen and progesterone receptors, normally considered as favourable prognostic factors, resulting in a well-represented group of patients as in the ACOSOG Z0011 trial. In our patients who developed LR, LRR and/or DM, the median age was 47 years (range 41–56 years). In 6 of 7 patients, Ki-67 expression was high with a median percentage of 35% (range 12–70%); only 1 patient had low Ki-67 expression (5%).

The prognostic value of Ki-67 was analysed regarding OS and DFS (in 25 and 29 studies, respectively) in the meta-analysis by Petrelli et al. A high Ki-67 cut-off level (at least 10%) resulted associated with more than 50% risk of death among early BC patients, particularly in those with ER + disease, and it was also associated with a greater risk of recurrence [25]. Therefore, Ki-67 represents an important prognostic factor and it could be considered also a predictive factor in terms of adjuvant therapy benefit in node-positive BC patients.

Our study has some limitations. First, our median follow-up is relatively short (50 months against 111 months of the ACOSOG trial). Considering our preliminary encouraging results, a prolonged follow-up will be necessary to confirm LR, RR, LRR, DM, DFS and OS good rates for patients treated with SLN and ALND omission.

Another limitation could be the lower percentage of patients with common negative prognostic factors, such as basal-like type neoplasms (triple-negative tumours), G3 tumour or lymphovascular invasion. Our positive survival results could have been influenced by endocrine therapy, in positive oestrogen and progesterone receptor status.

## Conclusion

We report our prospective observational study on 133 breast cancer patients with cT1‑2 cN0 invasive cancer and 1 or 2 positive sentinel lymph nodes, treated with breast conserving surgery and sentinel lymph node biopsy alone, and blinded planned tangential field radiotherapy without nodal irradiation. We achieved excellent local, regional and distant disease control. Our preliminary results suggest that axillary lymph node dissection (ALND) could be omitted, without nodal irradiation, sparing the patient from potential morbidity without increasing the risk of disease recurrence.
